# Fracture du corps caverneux de la verge

**DOI:** 10.11604/pamj.2020.37.272.21010

**Published:** 2020-11-25

**Authors:** Ahmed Ibrahimi, Idriss Ziani

**Affiliations:** 1 Service d´Urologie-A, Centre Hospitalo-Universitaire Ibn Sina, Faculté de Médecine et de Pharmacie, Université Mohammed V, 10000, Rabat, Maroc

**Keywords:** Fracture de verge, albuginea, corps caverneux, dysfonction érectile, Penile fracture, albuginea, corpus cavernosum, erectile dysfunction

## Abstract

Penile fracture is a rare urological emergency requiring immediate surgery, ideally in the first 24h, to prevent complications which are dominated by erectile dysfunction and penile curvature. The highest rates are reported in the Middle East and the Maghreb, most often secondary to false step in coitus or penile manipulations during erection such as the “taqaandan” maneuver. We report the case of a 34-year-old patient with no specific pathological history presenting to the Emergency Department with penile pain associated with swelling and deformity. The patient reported cracking sound during false step in coitus followed by acute penile pain with complete detumescence of the penis, with no associated urinary signs. Clinical examination revealed swollen penis, the seat of a hematoma, suggesting aubergine sign and a small painful induration on the dorsal side of the right corpus cavernosum on palpation. Emergency ultrasound confirmed the presence of a fracture line in the albuginea of the corpus cavernosum at 5 cm from the suspensory ligament of the penis. Direct approach based on elective incision of the induration was performed, which revealed longitudinal fracture measuring 18 mm in length involving the albuginea of the dorsal face of the right corpus cavernosum. After evacuation of hematoma, suture of the edges of the fracture line was performed with separate stitches of Vicryl 3-0. The post-operative suite was simple. At the follow-up visit the patient had recovered normal erectile function, with no induration or fibrosis at the level of the incision or penile curvature.

## Image en médecine

La fracture de la verge est une urgence urologique rare, elle doit être opérée dans les plus brefs délais, idéalement dans les premières 24h pour prévenir les complications qui sont dominées par la dysfonction érectile et la coudure de la verge; les taux les plus élevés sont rapportés au Moyen-Orient et au Maghreb, le plus souvent secondaire à un faux pas de coït ou aux manipulations de la verge en érection comme la manœuvre de « taqaandan ». Nous rapportons le cas d´un patient âgé de 34 ans, sans antécédents pathologiques particuliers qui s´est présenté aux urgences pour douleur pénienne associée à une tuméfaction et déformation de la verge. Le patient rapportait lors d´un faux pas de coït un claquement suivi d´une douleur pénienne aiguë avec détumescence complète de la verge, sans signes urinaires associés. L´examen clinique révélait une verge tuméfiée, siège d´un hématome donnant l´aspect typique de verge en «aubergine», avec présence à la palpation d´une petite induration douloureuse sur la face dorsale du corps caverneux droit; l´échographie réalisée en urgence a confirmé la présence d´un trait de fracture au niveau de l´albuginée du corps caverneux à 5 cm de ligament suspenseur de la verge. Notre patient a bénéficié d´un abord direct par une incision élective sur l´induration, qui a permis de mettre en évidence une fracture longitudinale de 18 mm de longueur intéressant l´albuginée de la face dorsale du corps caverneux droit; après évacuation de l´hématome, on a procédé à la suture des berges du trait de fracture par des points séparés au Vicryl 3-0. Les suites post-opératoires étaient simples, le patient a été revu en consultation de contrôle avec une fonction érectile normale, sans induration ou fibrose en regard de l´incision ni coudure de la verge.

**Figure 1:**
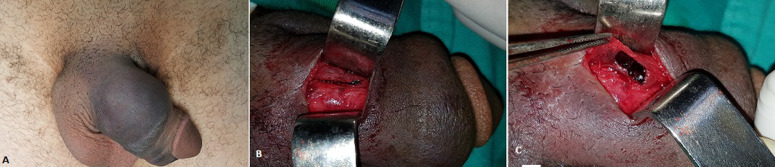
A) aspect de verge en aubergine suite à un faux pas de coït lors d´un rapport sexuel; B) trait de fracture longitudinal de l´albuginée de la face dorsale du corps caverneux droit; C) hématome secondaire à la déchirure de l´albuginée visible en peropératoire

